# Innovative Therapy for Alzheimer’s Disease-With Focus on Biodelivery of NGF

**DOI:** 10.3389/fnins.2019.00038

**Published:** 2019-02-05

**Authors:** Sumonto Mitra, Homira Behbahani, Maria Eriksdotter

**Affiliations:** ^1^Division of Clinical Geriatrics, Center for Alzheimer Research, Department of Neurobiology, Care Sciences and Society, Karolinska Institutet, Huddinge, Sweden; ^2^Division of Neurogeriatrics, Center for Alzheimer Research, Department of Neurobiology, Care Sciences and Society, Karolinska Institutet, Solna, Sweden; ^3^Aging Theme, Karolinska University Hospital, Karolinska Institutet, Stockholm, Sweden

**Keywords:** NGF, encapsulated cell biodelivery, Alzheimer disease, cholinergic, neurotrophins

## Abstract

Alzheimer’s disease (AD) is a progressive neurodegenerative disorder associated with abnormal protein modification, inflammation and memory impairment. Aggregated amyloid beta (Aβ) and phosphorylated tau proteins are medical diagnostic features. Loss of memory in AD has been associated with central cholinergic dysfunction in basal forebrain, from where the cholinergic circuitry projects to cerebral cortex and hippocampus. Various reports link AD progression with declining activity of cholinergic neurons in basal forebrain. The neurotrophic molecule, nerve growth factor (NGF), plays a major role in the maintenance of cholinergic neurons integrity and function, both during development and adulthood. Numerous studies have also shown that NGF contributes to the survival and regeneration of neurons during aging and in age-related diseases such as AD. Changes in neurotrophic signaling pathways are involved in the aging process and contribute to cholinergic and cognitive decline as observed in AD. Further, gradual dysregulation of neurotrophic factors like NGF and brain derived neurotrophic factor (BDNF) have been reported during AD development thus intensifying further research in targeting these factors as disease modifying therapies against AD. Today, there is no cure available for AD and the effects of the symptomatic treatment like cholinesterase inhibitors (ChEIs) and memantine are transient and moderate. Although many AD treatment studies are being carried out, there has not been any breakthrough and new therapies are thus highly needed. Long-term effective therapy for alleviating cognitive impairment is a major unmet need. Discussion and summarizing the new advancements of using NGF as a potential therapeutic implication in AD are important. In summary, the intent of this review is describing available experimental and clinical data related to AD therapy, priming to gain additional facts associated with the importance of NGF for AD treatment, and encapsulated cell biodelivery (ECB) as an efficient tool for NGF delivery.

## Introduction

The world’s population is aging at a great pace, especially in the western developed countries where mean life expectancy is usually high (>60 years) (World Health Organization [WHO], 2018). Increased life expectancy, owing to better medical and technological advancements, allows a person to gain higher achievements in life (professional, personal, social, etc.). However, increased age also brings in several age-associated health issues like sarcopenia, cancer, dementia, diabetes, cardiovascular diseases, hypertension, osteoporosis, and others, which become major reasons of sufferings (emotional and economical) for the affected patients and associated families (Franceschi et al., 2018). Table 1 summarizes the mean age of population and the percent of population above 60 years of age in the current top 10 countries in the world (U.S. News & World Report; CIA fact book; World Report on Ageing and Health 2015). Although several other countries have both lower mean age and percent of aged population (especially in Africa where typically less than 9% of the population is above 60 years old and forecasted to remain the same until 2050), but the usual life expectancy in those countries is also low due to several confounding factors (poverty, unmet medical situations, geo-political issues, etc.). This puts focus on developed countries and demands a well-planned approach to deal with the health burden of the aging population.

**Table 1:** Summary of population statistics in the current top 10 countries in the world.

Country	Mean age (years)	Mean age (male) (years)	Mean age (female) (years)	% Population above 60 years age (as of 2015)	% Population above 60 years age (2050 forecast)
Switzerland	42.4	41.4	43.4	20–24	30 or more
Canada	42.2	40.9	43.5	20–24	30 or more
Germany	47.1	46	48.2	25–29	30 or more
United Kingdom	40.5	39.3	41.7	20–24	30 or more
Japan	47.3	46	48.7	30 or more	30 or more
Sweden	41.2	40.2	42.2	25–29	25–29
Australia	38.7	37.9	39.5	20–24	25–29
United States of America	38.1	36.8	39.4	20–24	25–29
France	41.4	39.6	43.1	25–29	30 or more
Netherlands	42.6	41.5	43.6	20–24	30 or more


The World Health Organization (WHO) identifies Alzheimer’s disease (AD) (including other dementia’s) as the fifth leading cause of death worldwide in the year 2016 (∼2 million deaths), only after ischemic heart disease, stroke, chronic obstructive pulmonary disease (COPD) and lower respiratory infections (World Health Organization [WHO], 2016). The incidence of dementia related death has doubled since 2000 (ranked at the 14th spot), which is an important aspect of concern and can be attributed to higher life expectancy. Among the upper-middle-income and high-income countries, Alzheimer’s and dementia associated deaths were ranked at fifth and third spots, respectively. By 2050, the world’s population above the age of 60 years will nearly double (from 900 million in 2015 to 2 billion), among which 80% of them will reside in low- and middle-income-countries (World Health Organization [WHO], 2018). Therefore, immediate attention should be provided in developing strategies to prevent the occurrence of such devastating health conditions. However, until such measures are developed, palliative methods will remain of utmost importance to relieve the suffering and enhance quality of life for both the affected individual and the caregiver.

Currently almost 47 million people live with dementia, which is likely to become 131 million by 2050 (World Alzheimer Report, 2016). AD alone constitutes about 60–80% of dementia cases in North America and Europe, and by itself is the single largest cause of dementia related death worldwide (Puzzo et al., 2015). The global expense for the overall direct and indirect costs are estimated to be around 1.09% and 0.65% of the global gross domestic product (GDP), respectively, whereas in the United States, the cost of patients care is in the excess of United States $100 billion (Puzzo et al., 2015; World Alzheimer Report, 2016).

Alzheimer’s disease is a neuropathological condition in which the affected individual displays a varied constellation of cognitive impairments, which progressively hamper normal daily life activities. Pathologically, AD is identified by the presence of aggregated form of amyloid-β (Aβ) proteins (produced by β-cleavage of amyloid precursor protein, APP) as extracellular plaques and hyper-phosphorylated forms of tau protein as intra-cellular tangles. AD can be classified into two major groups, familial (early onset, Mendelian-inheritance) which has an occurrence rate of ∼5% and sporadic (late onset) with an occurrence rate of 95%, respectively (Lista et al., 2015). Apart from establishing some genetic basis of the disease as predisposing factors (ApoE4, APP, presenilin 1, presenilin 2) in case of familial AD, the overall cause of the sporadic AD remains multimodal and phenotypically heterogeneous. Recently several genome wide association (GWAS) studies resulted in the identification of more than 20 genetic loci as AD associated risk factors, which were related to various physiological processes including the immune system, inflammation, lipid metabolism, and vesicle recycling (Van Cauwenberghe et al., 2016). Among non-genetic determinants, several lifestyle factors (diabetes, smoking, physical inactivity, depression, diet, low education) and early life vascular diseases were identified as confounding factors in developing AD later in life (Qiu et al., 2010; Scheltens et al., 2016).

Among several factors, Aβ is one of the highly researched targets in the field of early onset AD neuropathology and is believed to be the causal factor of the disease. It has been more than 25 years since the proposal of the ‘amyloid hypothesis’ and many clinical trials have been directed toward this hypothesis to reduce Aβ levels in search of curing AD itself (Selkoe and Hardy, 2016). However, recent failures of several clinical trials targeting Aβ clearance have initiated interest in the search of alternative targets (De Strooper and Karran, 2016; Makin, 2018). Unless further scientific progress can be achieved to find an effective strategy to cure or halt the disease progression in AD, the current state of palliative care will remain as an important aspect in treating the present and future AD affected individuals.

Presently, the only available treatment option is the use of drugs called cholinesterase inhibitors (ChEIs, which includes donepezil, rivastigmine, and galantamine) and glutamate receptor (NMDA) antagonist (memantine). Activity of acetylcholinesterase in central nerve cells was observed a few decades back leading to large research efforts to therapeutically target them for modifications of the cholinergic pathways in AD (Giacobini and Zajicek, 1956; Marchi et al., 1980; De Sarno et al., 1989; Giacobini et al., 1989; Messamore et al., 1993a,b; Zhu and Giacobini, 1995; Giacobini, 1998, 2004; Taylor, 1998). These drugs provide symptomatic relief and temporarily stabilize cognitive functions, without any impact on disease progression or severity (Rodda and Carter, 2012; Deardorff et al., 2015). However, some studies have shown that ChEIs can modulate Aβ pathways by affecting Aβ generation and fibrillation properties (Inestrosa et al., 1996; Nordberg, 2006). The ChEIs were developed to halt the activity of the acetylcholine (ACh) degrading enzyme called acetylcholinesterase (AChE), which in turn is part of the cholinergic pathway, and was inherently based on the ‘cholinergic hypotheses’ of AD development (Bartus et al., 1982; Bartus, 2000; Mufson et al., 2008). The cholinergic hypothesis highlights the importance of the basal forebrain, which contains the cholinergic neurons that are important for normal cognition, and is found degenerated in AD (Bartus et al., 1982).

## AD and the Cholinergic System

The cholinergic system of the human brain has been attributed to be the master regulator of executive and mnemonic functions, and its loss is associated with cognitive decline (Ballinger et al., 2016; Gielow and Zaborszky, 2017). During the initial studies on AD related cognitive impairments in the late 1970s and early 1980s, several groups demonstrated the association between cholinergic neurons depletion in the basal forebrain and simultaneous cognitive decline (Drachman and Leavitt, 1974; Davies and Maloney, 1976; Smith and Swash, 1979; Whitehouse et al., 1982). These studies along with many other supporting observations led to the proposal of the cholinergic hypothesis, which for the first time proposed the importance of cholinergic neuron degeneration in AD associated cognitive impairment (Bartus et al., 1982). Several studies have reported discrepancies in this hypothesis, including the observations that the levels of the ACh-synthesizing enzyme (choline acetyltransferase, ChAT) actually increase during early stages of AD. Nevertheless, proponents of the theory attribute this change as a compensatory event linked to the early cholinergic deficit during AD initiation (Bartus, 2000; DeKosky et al., 2002; Mesulam, 2004; Mufson et al., 2008). Noteworthy, ChAT activity is not the rate-limiting step in ACh synthesis (Haubrich and Chippendale, 1977; Brandon et al., 2004), and the levels of ACh are maintained even when ChAT levels are depleted by 90% (Jenden et al., 1976). Widespread loss of cholinergic neurons in the basal forebrain during advanced stages of AD associated with incremental loss in cognition strengthened the cholinergic hypothesis and established the involvement of cholinergic loss in AD (Bierer et al., 1995; Bartus, 2000; Ferreira-Vieira et al., 2016; Hampel et al., 2018).

The reports on cholinergic deficits associated with early cognitive decline are supported by recent studies, where a long preclinical phase followed by mild cognitive impairment are stages in the AD development (Jack et al., 2012). Recent reports demonstrate a direct correlation between reduced basal forebrain volume and cognitive decline, which were found to be correlated with Aβ burden (Grothe et al., 2010; Kerbler et al., 2015). Interestingly, the decrement in the volume of basal forebrain precedes any major impact on hippocampal volume and predicts the cortical spread of AD pathology, thereby implying the early susceptibility of basal forebrain during AD development (Schmitz et al., 2016; Teipel et al., 2018). The cholinergic neurons are essentially dependent on the neurotrophic factor, nerve growth factor (NGF), for their survival and plasticity, and NGF metabolism is reported to be hampered in AD (Madziar et al., 2005; Iulita and Cuello, 2014; Isaev et al., 2017). Impaired signaling of NGF through its receptors is another important factor contributing to the development of AD (Latina et al., 2017).

## Physiological Role of NGF

Nerve growth factor is the founding member of the ‘neurotrophin family,’ primarily produced from the GABAergic neurons in the adult rat brain cortex among many other cell types across the brain (Lauterborn et al., 1995; Biane et al., 2014). NGF regulates differentiation, growth, survival and plasticity of certain cell types, including the cholinergic neurons, in the central and peripheral systems (Levi-Montalcini, 2004; Niewiadomska et al., 2011). NGF is tonically secreted in a precursor form (proNGF), which following maturation by the extracellular protease plasmin is converted to the mature form (mNGF). Both proNGF and mNGF are biologically active (Iulita and Cuello, 2014). The cholinergic neurons require mNGF for their growth and plasticity, and expresses the NGF receptors, tropomyosin receptor kinase A (TrkA, high affinity receptor) and p75 (also known as low-affinity NGF receptor, LNGFR; or p75 neurotrophin receptor, p75NTR), respectively (Niewiadomska et al., 2011; Isaev et al., 2017). Although both proNGF and mNGF can induce neurotrophic effects through TrkA (mNGF more potent than proNGF), but proNGF alone is also capable of inducing apoptotic signaling through its interaction with p75 (with cooperation from another receptor called sortilin) (Al-Shawi et al., 2007; Bradshaw et al., 2015; Ioannou and Fahnestock, 2017). Mature NGF is finally degraded by the matrix metalloproteinase 9 (MMP9) whose expression is upregulated in AD (Mroczko et al., 2013; Iulita and Cuello, 2014).

During normal physiological conditions, the cholinergic neurons in the basal forebrain play an important role in cognition through ACh innervation to the cortex and hippocampus. NGF is released by the postsynaptic cortical and hippocampal neurons, and is taken up by the pre-synaptic cholinergic neuronal projections through TrkA/p75 receptors (Segal, 2003; Biane et al., 2014). These receptor-bound-NGF molecules are then internalized and retrogradely transported to cholinergic cell bodies in the basal forebrain to initiate further signaling cascades that includes cell survival, growth and release of ACh through the cortico-hippocampal projections (DiStefano et al., 1992; Ure and Campenot, 1994; Campenot and MacInnis, 2004). During the pathological conditions of AD, metabolism of NGF has been found to be altered, resulting in an accumulation of proNGF levels and reduction of mNGF, which in rats have been shown to induce loss of cortical synapses and atrophy of the basal forebrain cholinergic neurons (Mufson et al., 1995; Niewiadomska et al., 2011; Iulita and Cuello, 2014). Widespread cholinergic cell loss is observed during severe stages of AD. However, in the earlier stages of AD, intact albeit functionally altered cell bodies along with a mild reduction in synaptic density have been found (Mesulam, 2004). Since these cholinergic cell bodies retain their sensitivity to NGF, delivery of NGF has been targeted over the years as a potent method to revive cholinergic signaling in cortex and hippocampus (*discussed later*).

## What Goes Wrong in AD in Relation to NGF?

**FIGURE 1:**
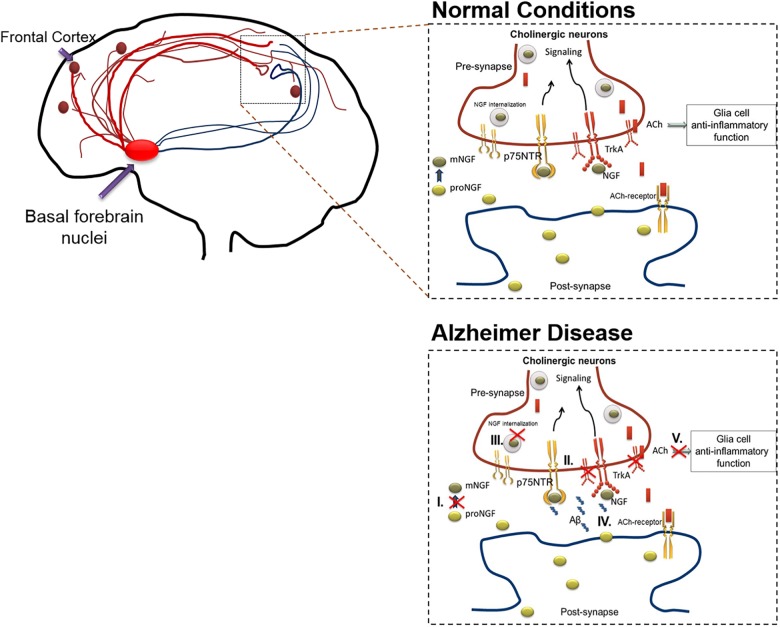
The action of cholinergic basal forebrain neurons in normal and in AD conditions. Cholinergic neurons are known to innervate the cortex and hippocampal regions of the brain. Under normal condition, postsynaptic resident neurons of cortical and hippocampal regions release proNGF, which is processed extracellularly (proNGF to mNGF) and taken up by the presynaptic cholinergic neurons (through TrkA, predominantly). The mNGF is then retrogradely transported within the cholinergic neurons, to the cell nuclei situated in the basal forebrain, to initiate gene expression and signaling cascades. These signaling cascades facilitate the release of acetylcholine from the cholinergic terminals present in cortical or hippocampal regions, thereby initiating cholinergic signaling in these brain regions, which includes anti-inflammatory effects mediated through alpha-7-nicotinic receptors in the glial populations. As mentioned below, this cross-talk is altered during AD in various ways which results in cholinergic degeneration. The figure illustrates that the cholinergic system is affected due to various pathways including the following: **(I)** Altered NGF maturation, **(II)** Skewed TrkA/p75 receptor ratio, **(III)** Inefficient axonal transport and signaling, **(IV)** Aβ induced modulation of NGF receptors. Apart from inducing death, increased Aβ levels can interact with several NGF receptor types including, alpha-7-nicotinic, and metabotropic receptors, leading to increased inflammation and signaling impairments. **(V)** Sub-optimal release of acetylcholine leading to glial activation and subsequent inflammatory response. These alterations compromise the availability of mNGF to the basal forebrain cholinergic neurons, which in-turn culminates in hampered cholinergic innervation to cortex and hippocampal regions of the brain. The red crossed out areas represent the affected pathways. Aβ, amyloid β; ACh, acetylcholine; AD, Alzheimer’s disease; NGF, nerve growth factor; TrkA, tyrosine receptor kinase A.

In AD, the cholinergic system is affected due to various processes including the following: (1) Altered NGF maturation, (2) Skewed TrkA/p75 receptor ratio, (3) Inefficient axonal transport and signaling, (4) Aβ induced modulation of NGF receptors, (5) Suboptimal ACh innervation induced inflammatory response, and (6) Aβ cytotoxicity. The collective interplay of these factors are depicted in Figure 1.

During the initiation and progression of AD pathology, the declining function of cholinergic innervation to cortex and hippocampus occurs due to inefficient maturation of NGF. This is attributed to reduced production and elevated clearance of mNGF (Cuello and Bruno, 2007; Allard et al., 2012) as well as reduction in levels of TrkA-receptors in neurons in nucleus basalis and cortex (Mufson et al., 1997) affecting the retrograde transport of NGF (Mufson et al., 1995). Simultaneous evaluation demonstrated hampered plasmin machinery along with increased MMP9 activity, culminating in elevated levels of proNGF in the brain tissue (Fahnestock et al., 2001; Peng et al., 2004; Fabbro and Seeds, 2009; Mroczko et al., 2013). Higher proNGF levels not only induce pro-apoptotic signaling, but are also found to affect the receptor binding of mNGF and its axonal trafficking and result in retrograde atrophy of cholinergic neurons in the basal forebrain (Sobottka et al., 2008; Ioannou and Fahnestock, 2017; Allard et al., 2018). The apoptotic signaling induced by pro-NGF is dependent on the relative expression of TrkA and p75, and is favored by a decreased ratio of TrkA/p75 that is also evident in AD (Counts et al., 2004; Ginsberg et al., 2006; Masoudi et al., 2009). These alterations have been shown in AD-like mice models (Tiveron et al., 2013). Interestingly, using the AD11 mice model that expresses anti-mNGF antibodies specifically in the brain, it was observed that lack of mNGF leads to early inflammation and Alzheimer’s neurodegeneration (Capsoni et al., 2011). NGF was found to have a direct role in modulating microglial cells toward a non-inflammatory phenotype (Rizzi et al., 2018).

In this regard, a significant cross talk between NGF and the Aβ generation machinery is present where Aβ is reported to hamper NGF maturation and NGF in turn can reduce Aβ generation, while plasmin is known to degrade Aβ (Tucker et al., 2000; Bruno et al., 2009; Yang et al., 2014). Similarly, NGF can affect APP phosphorylation levels thereby dictating Aβ generation whereas APP can modulate NGF receptor trafficking and axonal transport thereby affecting NGF downstream signaling (Zhang et al., 2013; Triaca and Calissano, 2016; Xu et al., 2016). It has been speculated that during aging, alteration in NGF maturation and signaling pathways can lead to increased Aβ generation and reduced clearance (Gutierrez-Fernandez et al., 2007; Matrone et al., 2008; Triaca and Calissano, 2016; Canu et al., 2017). Apart from inducing death, increased Aβ levels can interact with several receptor types including p75 (Yaar et al., 1997; Chakravarthy et al., 2010; May et al., 2017), alpha-7-nicotinic (Khan et al., 2010; Ni et al., 2013; Liu et al., 2017), and metabotropic receptors (Joseph and Fisher, 2003; Janickova et al., 2013), leading to increased inflammation and signaling impairments (Conejero-Goldberg et al., 2008; Mura et al., 2012; Ardura-Fabregat et al., 2017). Aβ can also impair choline re-uptake from the synaptic cleft thereby affecting ACh production in presynaptic neurons and alter ACh release leading to attentional deficits (Fu and Liu, 1997; Parikh et al., 2014). Since ACh modulates the cholinergic anti-inflammatory pathways in the glial population, hampered ACh release ensues initiation of inflammation (Wang et al., 2003; de Jonge and Ulloa, 2007; Treinin et al., 2017; Bagdas et al., 2018). Thus, it is important that mNGF levels are maintained to affect cholinergic signaling which in turn is responsible to maintain an anti-inflammatory tone in the neurological system (Pavlov et al., 2003).

In line with these findings obtained from the last three decades, NGF has been targeted to modify the associated signaling pathways and envisaged as a therapeutic strategy in AD.

## Therapeutic Interventions of NGF in AD

### Various Methods of NGF Delivery and Their Outcome

As a therapeutic factor, NGF has been utilized in clinical trials of varied diseases including peripheral neuropathies, human immunodeficiency virus infections, e.g., HIV and central nervous system’s (CNS) diseases like AD (Aloe et al., 2012). One of the most clinically relevant characteristic of the small neurotrophic factors like NGF is that this protein does not normally pass through the blood–brain barrier (BBB) (Liu and Chen, 2005; Wu, 2005). Furthermore, it is known that a direct delivery of neurotrophic factors can have serious peripheral side effects (Thoenen and Sendtner, 2002). These characteristics have resulted in shortcomings of systemic delivery of neurotrophic factors such as NGF in clinical trials for AD and neurological disorders.

## Direct Intra-Cerebral Infusion of NGF

As mentioned above, the essential problem for many molecules and compounds is insufficient diffusion across the BBB. Therefore, it becomes imperative for these molecules, or other therapeutic targets, to be delivered locally into the brain. One of the possible routes of local delivery methods is the local cerebral injection. The challenge of this method is to find effective and compatible doses of physiologically related amounts/volumes of every individual compound, which are needed to be tested. Additionally, the infusion may not remain in the target location long enough to elicit an effect, revealing another disadvantage of this method of drug delivery. Nevertheless, the local delivery of many compounds into the brain might be the only way to generate therapy for many neurodegenerative disorders.

Researchers and companies have developed the intra-cerebral infusion platform and the devices are commercially available for many research applications. The ALZET osmotic pumps are small infusion pumps that ensure constant delivery of compounds or proteins into the brain regions of interest. The pump is connected to a metal catheter, with a plastic platform that can be customized for targeting different brain regions or be adjusted in size for different animal models. The pumps exist in different sizes suitable for both animal and human CNS and can be utilized from a few hours to 28 days. The disadvantage of the pump infusion is its short duration of compound delivery when considered for a clinical setting. To minimize the risk for infection, the pump can be placed under the skin. At the end of the infusion period, the test reagent can be retrieved from the pump to determine the remaining biological activity. Intra-cerebral infusion has been a challenging route which has been perused in clinical trials involving AD patients (Hagg, 2007). As mentioned previously, AD patients show extensive cholinergic neuron loss that may be sensitive to the levels of mature NGF. Several experimental studies in aging-related animal models have found that NGF treatment improved cognitive ability in those aged animals showing cholinergic loss and dysfunction (Fischer et al., 1987; Hefti et al., 1989).

Our research group has previously performed a small clinical study in three AD patients infusing mouse 2.5S NGF into the cerebral lateral ventricle (Olson et al., 1992; Eriksdotter Jonhagen et al., 1998). The results from this study showed an increase in nicotinic receptors expression and regional cerebral blood flow in the neocortex following NGF-infusion, as analyzed by positron emission tomography (PET) imaging. This effect was maintained for several months until the NGF infusion was stopped. However, due to induced neuropathic pain and weight loss, the intracerebroventricular NGF infusion was terminated prematurely (Eriksdotter Jonhagen et al., 1998). When we used NGF infusion into the cerebral lateral ventricle, we were aiming to influence the entire area of cholinergic terminals. The lateral ventricles are the two largest cavities of the ventricular system of the human brain containing cerebrospinal fluid (CSF). Consequently, the CSF flows directly from the ventricles into the brain tissue and passes through the spaces between the cells where it eventually enters the subarachnoid space. Taking advantage of this normal feature that occurs in the brain, it is believed that the NGF infusion into the brain can be absorbed by tissues in the brain regions like cerebral cortex (Greitz and Hannerz, 1996). This feature provides a direct administration of NGF into the lateral-ventricle-containing CSF to reach the cortical sites where the improved function of cholinergic neurons is essential to counter cognitive decline.

However, the direct intracerebroventricular NGF administration caused pain in animals by affecting the dorsal root ganglion and hypothalamus function that is correlated to the pain response, and showed weight loss among treated animals (McKelvey et al., 2013). NGF delivery also indirectly affected the release of pain mediators including histamine, and prostaglandins from mast cells (Kawamoto et al., 2002). Interestingly these cells are capable to release NGF that might form a feedback loop that sensitizes adjacent nociceptive neurons (Kawamoto et al., 2002). In contrast, other animal studies have shown that when NGF is infused directly into the rat brain parenchyma (Hao et al., 2000) and primates (Tuszynski et al., 1990) no pain-related effects were observed.

## Peripheral Administration of NGF Using Nasal or Intraocular Delivery

Intranasal delivery (IND) method has overcome the obstacle for systemic delivery of both oral and parenteral drug routes into the brain. The nasal delivery of drugs provides a rapid absorption and delivers the drug directly into the brain via the olfactory route. The advantage of IND is the olfactory pathway which provides a non-invasive route for drug delivery directly to the brain, but does not protect from adverse effects. This approach has been utilized to deliver NGF into rodent brain (Cattaneo et al., 2008). The efficiency of intranasal administration was demonstrated in the AD11 anti-NGF transgenic mice model, showing reversion of the cognitive loss in AD11 mice (De Rosa et al., 2005). NGF eye drops using the ocular surface route has also been used to affect cholinergic markers in the basal forebrain of rats (Lambiase et al., 2007). Furthermore, ocular NGF has been able to activate the proliferation of cells in the sub-ventricular zone by showing increased layers of ki67 positive cells (marker for proliferative cells) which also were expressing p75NTR (Tirassa, 2011). However, this method has not been used in well-established animal models of AD. The intraocular delivery of NGF appears to be less characterized compared to the IND, particularly in terms of mechanisms and anatomical delivery to the brain (Dhuria et al., 2010). There has been studies utilizing the IND of mutated human NGF (R100) in an AD animal model (Capsoni et al., 2011, 2012), where the mutated human NGF retained the potential of NGF’s neurotrophic effect without eliciting the pain-related response. Surprisingly, such a test has not yet been validated in preclinical studies for neurodegenerative diseases (Capsoni et al., 2012). There have been various strategies for the delivery of any drug via IND such as nanotechnology-enabled drug delivery (e.g., lipid nanoparticles) for many diseases like cancer, technology of microencapsulation and the use of mucoadhesive materials and devices. However, despite the improvement and development of these systems, very few IND systems targeting AD have yet shown sufficient clinical relevance.

## Stem Cell-Mediated Delivery of Therapeutics

The use of stem cells for targeting tissues or organs of interest has received much attention as carriers for therapeutic agents. The definition of these cells is based on their capability of self-renewing and differentiation into specialized progeny according to their degree of potency (Mimeault et al., 2007). Stem cells can deliver neurotrophins into diseased brain, modulate endogenous synaptic plasticity, and enhance neuronal survival. High levels of neurotrophins such as brain derived neurotrophic factor (BDNF) and NGF are expressed in neural stem cells (NSCs) (Joseph and Fisher, 2003; Kamei et al., 2007; Dhuria et al., 2010). Interestingly, increased synaptic density in hippocampus, reversal of memory and neuronal loss in transgenic AD animal models after NSCs transplantation have been shown (Joseph and Fisher, 2003; Bentz et al., 2007; Kamei et al., 2007; Yamasaki et al., 2007; Blurton-Jones et al., 2009; Kim et al., 2010).

In spite of the potential clinical applications using stem cells, there is a need to understand the pathological changes in the brain of neurodegenerative disorders that might affect the transplanted stem cells. This method should be combined with the control and observation of migratory patterns of transplanted stem cells in animal models prior to clinical trials on humans. In order to use stem cells as a replacement strategy in AD, several issues have to be addressed. By *in vivo* and *ex vivo* studies, the migration of stem cells in different brain regions and areas should be tested. Detailed knowledge of the migration, differentiation and maturation of stem cells into various neuronal subtypes is needed. These neurons would then have to re-innervate the correct target and establish neuronal connections mimicking the normal brain circuitry. Because of the safety issues, the protocols for pre-clinical experiments should be carefully controlled, standardized and undergo extensive evaluation before initiation of clinical studies. Inflammation can cause and change the pathological environment in the brain, therefore there is a possibility that transplantation of stem cells may alter the inflammatory responses in the brain. A study by Lee et al. (2012), showed an influence on inflammatory response and pathogenesis in AD animal models, when they used NSCs and mesenchymal stem cells (MSCs) as a therapeutic choice. Therefore, studies are needed to understand the mechanisms involved in direct or indirect effects of stem cell transplantation in altering the inflammation caused by tissue injury or any kind of xenotransplantation. Studies of stem cell transplantation in immune-incompetent AD models would be interesting in order to elucidate this important question (Chen and Blurton-Jones, 2012). Another benefit for AD would be the NSCs mediating delivery of enzymes such as neprilysin to degrade Aβ (de Backer et al., 2010).

Survival and differentiation of NSCs may be influenced by immune responses and the pathology of the disease may affect the efficacy of stem cell mediated therapy. Thus, further studies are needed to show if AD-associated pathology can be involved in NSC survival and differentiation. Neuronal replacement has hitherto not been clinically successful for neurodegenerative disorders like AD (Chen and Blurton-Jones, 2012). Nevertheless, the positive outcome of patient-derived induced pluripotent stem cells (iPSCs) as a model of human genetic disorders (Grskovic et al., 2011), and reprogramming of the induced NSCs (iNSCs) from AD patients can be useful for such purposes. Two different reports presented the first steps of AD iPSCs as a potential route of AD therapy (Yagi et al., 2011; Israel et al., 2012). Collectively these data suggest that stem cell mediated therapy in AD could be beneficial, and further investigations on embryonic, neural and iPSCs will contribute a basis for a future therapeutic approach for AD.

## NGF Delivery Using Viral Vectors

Since the cholinergic system of the human brain is involved in memory function, and its loss is associated with cognitive decline, local NGF delivery directly to the cholinergic basal forebrain would be preferred. However, it poses a clinical and technical challenge.

The essential core of regenerative medicine revolves around cell therapy. In association with cell therapy utilization, viral vector-mediated gene transfer techniques, in particular those techniques developed for lentiviruses, have demonstrated some useful features. Hohsfield et al. (2013), demonstrated that infection by a lentiviral vector, which overexpressed NGF, showed successful production of effective NGF secretion. In parallel with these findings, lentivirus NGF gene delivery to the cholinergic basal forebrain for 1-year in aged monkeys showed no systemic leakage of NGF or formation of anti-NGF antibodies, nor activation of inflammatory markers in the brain or pain or weight loss (Nagahara et al., 2009a).

The first study using gene therapy in patients with AD was published in 2005 (Tuszynski et al., 2005). In this study, NGF gene delivery was performed to individuals with a mild AD diagnosis where the transfer of the NGF gene through genetically manipulated autologous fibroblasts was implanted into the basal forebrain. Data from this 2-year, open-label study, showed a significant increase of glucose uptake in several cortical regions and cognitive tests showed scores that were stabilized or declined at a slower rate than expected (Tuszynski et al., 2005, 2015). Also adeno-associated virus (AAV)-based gene delivery has been used in the brain of AD patients for the delivery of NGF in order to treat AD symptoms and progression (Rafii et al., 2014). A phase II clinical trial using AAV vectors expressing human NGF was recently published. This study included 49 AD patients randomly assigned to receive intracerebral injections of AAV-NGF or sham surgery. AAV-NGF delivery was well-tolerated over 2 years but with no effects on clinical outcomes. However, confirmation of accurate gene targeting by pathology is needed (Rafii et al., 2018).

There are certain limitations with *in vivo* gene therapy, particularly the permanent genetic modification of the patient’s brain cells and the obvious inability to control and stop the targeted release. However, with the development of self-inactivating viral vectors (SIV’s), the expression of transgenes can be delivered with minimally invasive surgery and can also be switched off (Kraunus et al., 2004; Schambach et al., 2007; Thornhill et al., 2008). Conversely, considering current technological platforms, using SIVs for long-term delivery of therapeutic proteins (for months or years) would still be a challenge, which is otherwise desirable for treating neurodegenerative disorders.

## Small Molecule Modulators of NGF and Its Receptors

Apart from utilizing the full-length NGF molecule as a therapeutic agent, several chemical compounds as well as peptides are exploited and developed which are termed NGF mimetics. These compounds/peptides resemble NGF epitopes and can interact with the NGF receptors to induce downstream signaling pathways or may also act as antagonists, thereby modulating neurotrophic signaling. Nevertheless, before using these kind of analogs as neurotrophic therapeutic molecules, similar varieties were initially developed to understand NGF and TrkA interaction, and also to detect and diagnose oncogenic transformation *in vivo* where TrkA levels were found to be overexpressed (Longo et al., 1990; LeSauteur et al., 1996). Toward the end of the 20th century, several groups reported the development of several classes of neurotrophin mimetics targeting different receptor types including TrkA modulators, p75 modulators and BDNF (LeSauteur et al., 1995; Spiegel et al., 1995; Estenne-Bouhtou et al., 1996; Longo et al., 1997; O’Leary and Hughes, 1998).

With the advent of time, several specific small molecule modulators have been developed which can selectively target specific neurotrophin receptors as shown in Table 2. Dr. Longo and his team have received success utilizing a small molecule mimetic of NGF targeting the p75 receptor, termed LM11A-31, showing reduced microglial activation after treatment in AD-like mice (James et al., 2017). The first clinical trial using small molecule mimetics with LM11A-31 is currently underway for treatment of mild to moderate AD patients (ClinicalTrials.gov Identifier: NCT03069014).

**Table 2:** An up-to-date comprehensive report on the type and class of different small molecule modulators evaluated so far, targeting various neurotrophin receptors in different *in vitro* and *in vivo* models.

Class	Type	Name	Model	Outcome	Reference
NGF	Dipeptide	GK2	*In vitro*	Neuroprotection	Antipova et al., 2011; Gudasheva et al., 2015; Stelmashook et al., 2015
			Stroke (ischemic and hemorrhagic)	Infarct reduction, anti-amnestic, improved cognition	Seredenin et al., 2011a,b; Kraineva et al., 2013; Povarnina et al., 2017
			Alzheimer’s disease	Improved cognition	Povarnina et al., 2013
			Diabetes	Anti-hyperglycemia, improved cognition	Ostrovskaya et al., 2017
			Parkinson’s disease	Improved behavioral outputs, neuroprotection	Povarnina et al., 2011
			Traumatic brain injury	Neuroprotection	Stelmashook et al., 2015
		P92	*In vitro*	Neurite outgrowth, cell signaling	Xie et al., 2000
		GTS-113	*In vitro*	Neuroprotection against oxidative stress	Gudasheva et al., 2017b
			Stroke (ischemic)	Neuroprotection, induces hyperalgesia	Gudasheva et al., 2017b
		GK6	*In vitro*	Neuroprotection	Gudasheva et al., 2015
	Peptidomimetic	NL1L4	*In vitro*	Neuronal differentiation and TrkA activation	Colangelo et al., 2008
		L1L4	*In vitro*	Neuronal differentiation and TrkA activation	Colangelo et al., 2008
			Peripheral neuropathic pain	Pain reduction, neuronal function recovery	Colangelo et al., 2008
		BB14	Peripheral nerve injury	Reverses allodynia and hyperalgesia, reduces gliosis	Cirillo et al., 2012
		C(92–96)	*In vitro*	TrkA activity and neuroprotection	LeSauteur et al., 1995; Maliartchouk et al., 2000a
		5C3 and/or MC192 Fabs	*In vitro*	TrkA activity and neuroprotection	Maliartchouk et al., 2000a
		D3	*In vitro*	Differentiation of primary cells	Maliartchouk et al., 2000b
			Age associated cognitive impairment	Improved behavioral outputs, neuroprotection	Bruno et al., 2004
			Alzheimer’s disease (transgenic APP mice)	Improved spatial learning and long-term memory	Aboulkassim et al., 2011
			Rat model of dry eye (scopolamine induced)	Improves quality and stability of tear film, improves healing	Jain et al., 2011
	Non-peptidic	MT2	*In vitro*	Survival and differentiation, neuroprotection	Scarpi et al., 2012
					
BDNF	Small molecule partial agonist of TrkB	LM22A-4	Rett syndrome	Improves respiratory function, synaptic plasticity	Schmid et al., 2012; Kron et al., 2014; Li et al., 2017
			Non-arteritic Anterior Ischemic Optic Neuropathy	Enhanced cell survival	Ali Shariati et al., 2018
			Stroke (hypoxic-ischemic)	Functional recovery, neurogenesis	Han et al., 2012
			Huntington’s disease (R6/2 and BACHD mice)	Normalization of signaling pathways, cognition, and dendritic spine density	Simmons et al., 2013
			*In vitro*	Neuroprotection, improved cell signaling	Massa et al., 2010
			Traumatic brain injury	Improved motor learning, neuroprotection	Massa et al., 2010
			Epilepsy	Decreased epileptiform discharges, increased cell functionality	Gu et al., 2018
			Alcohol abuse disorders	Modulates alcohol intake (reduce)	Darcq et al., 2016; Warnault et al., 2016
		TDP6	Pharmacological animal toxicity model (cuprizone)	Re-myelination, TrkB activation and signaling	Fletcher et al., 2018
	Flavonoid	7,8-dihydroxyflavone	*In vitro*	Neuroprotection	Tsai et al., 2013; Liu et al., 2014
			Alzheimer’s disease (5xFAD mice)	Reduces memory deficit and BACE1 expression	Devi and Ohno, 2012
			Multiple	Neuroprotection	Jang et al., 2010
	TrkB and TrkC co-activator	LM22B-10	*In vitro*	Neurite outgrowth	Yang et al., 2016
			Aged mice	Elevated hippocampal dendritic spine density	Yang et al., 2016
	Dimeric dipeptide	GSB-106	Depression	Have anti-depressant effect	Kudrin et al., 2012; Seredenin et al., 2013; Gudasheva et al., 2016; Gudasheva et al., 2017a
			Stroke	Recovery, reduced infarct size, cell signaling	Gudasheva et al., 2016
					
p75	Small molecule negative modulator	LM11A-31	Traumatic brain injury	Neuroprotection, improved cognition, reduced glial activation	Shi et al., 2013
			*In vitro*	Neuroprotection, reverses synaptic impairment	Massa et al., 2006; Yang et al., 2008; Shi et al., 2013
			Status Epilepticus	No significant effect	Grabenstatter et al., 2014
			Alzheimer’s disease (Thy-1 hAPPLond/Swe (APPL/S) and Tg2576)	Cholinergic neurite dystrophy reversal; reduces microglial activation, Tau phosphorylation and memory impairments	Knowles et al., 2013; Nguyen et al., 2014; Simmons et al., 2014; James et al., 2017
			Experimental peripheral neuropathy	Normalization of signaling pathways, reversal of nerve injury	Friesland et al., 2014
			Huntington’s disease (R6/2 and BACHD mice)	Normalization of signaling pathways and cognition, extended survival, reduced microglial activation	Simmons et al., 2016, 2018
			Spinal cord injury	Normalized motor function, improved myelination and oligodendrocyte survival	Tep et al., 2013
			HIV associated neuro-pathogenesis	Normalized calcium signaling, mitochondrial function and movement	Meeker et al., 2012; Meeker et al., 2016
		LM11A-24	AβPP(L/S) transgenic mice	Reduces Tau phosphorylation, microglial activation and memory impairments	Nguyen et al., 2014
			*In vitro*	Neuroprotection, reverses synaptic impairment	Massa et al., 2006; Pehar et al., 2006; Yang et al., 2008
	Cyclic peptidergic modulator	P7	*In vitro*	Enhances survival, no neurite outgrowth, blocks Abeta-p75 interaction	Longo et al., 1997; Yaar et al., 2007
					
CNTF	Peptide mimetic (tetrameric)	Peptide 021	Alzheimer’s disease (3 × Tg)	Rescue synaptic/cognitive deficits, neurogenic, decreased tau accumulation, BDNF expression, neuroprotection	Kazim et al., 2014; Baazaoui and Iqbal, 2017a,b
			Aging	Restores synaptic deficits and metabolic profile, reduces CSF tau levels	Bolognin et al., 2014; Khatoon et al., 2015
			Down Syndrome (Ts65Dn mouse)	Rescue developmental delays, memory impairments	Kazim et al., 2017


## Cell Mediated NGF Delivery

**FIGURE 2:**
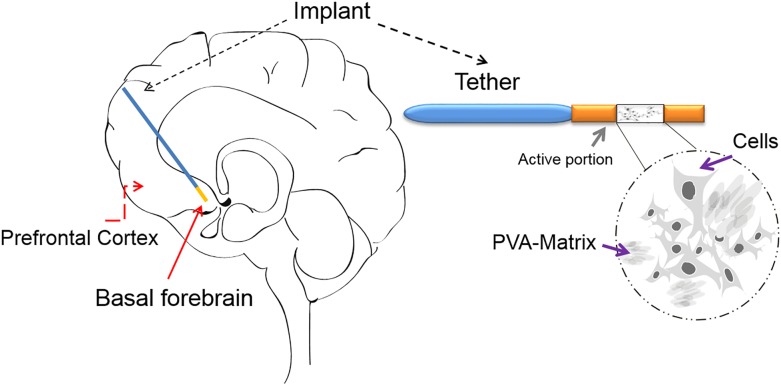
The ECB device: The ECB consists of a catheter-like device, which has approximately an 11 mm-long active portion and an outer diameter of 0.72 mm. In the active portion, the genetically modified human cell line (ARPE-19) is depicted growing on the spongy polyvinyl alcohol (PVA) cylindrical scaffold/matrix (a sagittal view is shown), within the semi-permeable polyethersulfone hollow fiber membrane, which allows for the influx of nutrients and the efflux of mature NGF. The membrane is in-turn linked to an inert polyurethane tether, which at its other end is finally attached to the edge of the burr hole. The device can be implanted with precision using a custom-made frame adapter, fitting a standard stereotactic frame. The schematic drawing of an implanted ECB device shows the active portion in orange and the tether in blue.

An alternative and reliable approach to deliver therapeutic molecules directly to the brain region of interest is the encapsulated cell biodelivery (ECB) system (Figure 2).

Lim and Sun (1980) achieved one of the earliest attempts to develop ECB in 1980, utilizing cross-linked alginate membranes while trying to deliver islets in rats. Subsequent studies employed ECB biodelivery of various neurotrophins including NGF, CNTF, and other sources including dopamine releasing cells and chromaffin cells (Emerich et al., 1994; Aebischer et al., 1996; Buchser et al., 1996; Winn et al., 1996; Lindner and Emerich, 1998). In the forthcoming years, several groups attempted to deliver a variety of neurotrophins like glial cell line-derived neurotrophic factor (GDNF), ciliary neurotrophic factor (CNTF), NGF, BDNF, meteorin, galanin, and cometin. To achieve this, various encapsulation materials like alginate, poly (acrylonitrile-co-vinyl chloride) copolymer (PAN-PVC), alginate-poly-L-ornithine-alginate, poly(acrylonitrile), poly(sulfone), poly(ether sulfone) (PES), poly(amides), poly(propylene), and poly(ethylene), hydrogel, nano-carriers and agarose/poly(styrene sulfonic acid) mixed gel were employed (Orive et al., 2004, 2015; Zanin et al., 2012; Angelova et al., 2013; Emerich et al., 2014; Govender et al., 2017). More recently, 3D printing technologies are being used to construct advanced, highly defined microstructures for drug delivery (Son et al., 2017).

The ECB technology aims to integrate the therapeutic potency of gene therapy along with the precision of a safe retrievable device (Lindvall and Wahlberg, 2008). The capsule comprises a biocompatible outer membrane and an inner core comprising cells, which are engineered to secrete therapeutics, for example a biologically active compound or coding for a polypeptide or a siRNA (Lindvall and Wahlberg, 2008). The modified cells are ensheathed inside a semi-permeable polymer based capsule, which is biologically inert, thereby not eliciting xenograft responses from the surrounding cells. These retractable capsules can be implanted into specific brain locations and can be retracted later to avoid complications (if needed). The semi-permeable membrane protects the engineered cells from host immune system while simultaneously allowing bi-directional free exchange of nutrients, oxygen and therapeutic products by diffusion across the capsule walls. The advantage of this method is based on the possibility of the semi-permeable capsule to deliver therapeutic entities in a local and controlled fashion, which cannot be administered orally or intravenously. Furthermore, the therapeutic molecule can be delivered at the desired site with high concentration thereby minimizing off-target side effects and complications. The semipermeable membrane functions for; (1) the efflux of the therapeutic protein, (2) protection of cells for immunogenic reactions, and (3) influx of nutrients from the host brain (Winn et al., 1996; Lindvall and Wahlberg, 2008). Therefore, this approach tackles previous drawbacks in NGF brain delivery (BBB passage, local delivery).

## Clinical Trials Using ECB-NGF Delivery and Initial Results in AD

Recently, the ECB platform was developed by NsGene Inc., with the intention to administer mature human NGF into the basal forebrain of AD patients, in an attempt to rescue basal forebrain cholinergic neurons from degeneration.

The first generation of device or implant, which is 11 mm in length, carries a genetically modified human retinal pigment epithelial cell line (ARPE-19) growing within the spongy polyvinyl alcohol scaffold, which is present inside the polyethersulphone hollow fiber membrane (Winn et al., 1996; Lindvall and Wahlberg, 2008). The semi-permeable membrane of the implant allows the influx of nutrients and the efflux of mNGF. Preclinical research from this study demonstrated that devices that were implanted in the basal forebrain of minipigs over a period up to 12 months, were retrieved without observing any associated complications (Fjord-Larsen et al., 2010). Implants showed to be intact and contained viable cells after explantation, whereas tissue in which the implant was localized showed increased levels of NGF (Fjord-Larsen et al., 2010). As compared to previous *ex vivo* and *in vivo* gene therapy studies, the ECB-NGF implant could be removed easily and thereby the treatment could be controlled or stopped whenever required.

Previously, our group utilized the ECB device in a pilot study involving ten AD patients. The study was performed in two parts in which the ECB device with NGF-producing cells were implanted into the basal forebrain of the AD patients. In the first part, six AD patients received the first generation implant having polyvinylalcohol foam scaffolding for 12 months (Eriksdotter-Jonhagen et al., 2012; Wahlberg et al., 2012). Analyses revealed that the result of this study was promising and therefore the study was continued to a second part using second generation of improved NGF releasing device containing polyethylene terephthalate (PET) yarn in additional 4 AD patients for 6 months of implantation (Eyjolfsdottir et al., 2016). The second part was carried out with improved NGF-secreting ARPE-19 cells which were transfected using the sleeping beauty transposon technology (Fjord-Larsen et al., 2010; Izsvak et al., 2010). The PET yarn improved cell viability and NGF release while also making the device manufacturing process more reproducible. In both part I and II, the devices were successfully removed after 12 and 6 months, respectively.

The outcome from both studies showed that the patients’ responses to the NGF-treatment varied and approximately half of the patients responded to the ECB-NGF-treatment with increased cholinergic markers (e.g., ChAT activity) in CSF correlating to improved cognition and brain glucose metabolism (Karami et al., 2015), less brain atrophy (Ferreira et al., 2015) and normalization of the EEG-pattern (*unpublished data*). In the second patient cohort in part II, we demonstrated safety and tolerability of the dose escalation from the second generation of ECB-NGF device and showed good cell survival and sustained NGF release from the encapsulated cells after explantation (Eyjolfsdottir et al., 2016).

Despite the encouraging results from ECB-NGF therapeutic approach, variations in the levels of NGF-release between capsules were observed. Furthermore, after testing for cell survival following capsule retrieval from AD patients, a few implants showed degenerating cells. Therefore, one of the challenges using this method is to understand the possible factors among all recipients that can affect secretion and survival of the encapsulated cells. Furthermore, in order to enable such approach, it is necessary to streamline and standardize the procedures. A careful characterization of the transplanted genetically engineered cells and of the quality and quantity of its produced therapeutic molecule is critical. To further develop the therapeutic platform, multifunctional approaches is needed to achieve optimal efficacy of the NGF-producing cells by investigating what factors are involved in influencing NGF-production.

Also the surgical procedure in association with the ECB implantation into the brain may cause small tissue injuries such as damaging small blood vessels, neurons and glial cells and the BBB (Hovens et al., 2012). The injured tissues surrounding the capsules might activate microglia and astrocytes which may respond by releasing various cytokines and free radicals (Feng et al., 2017; Xu et al., 2017), which in-turn can diffuse through the capsule membrane and affect the NGF-producing cell survival and NGF-release, respectively. Therefore, to some extent, the insertion of the ECB-NGF in the basal forebrain of the AD patients may lead to disruption of the normal brain environment. To test this hypothesis, the NGF-producing cells were examined by our group and was found to be negatively affected by IL-1β in a dose dependent manner suggesting that inflammation has a negative effect on the ECB cells (Eriksdotter et al., 2018). Moreover, Aβ peptides represent an obvious factor that might affect survival of the NGF-producing cells. Interestingly, at physiological concentrations, neither Aβ_40_ nor Aβ_42_ had any major impact on the NGF-release nor on the cell survival (Eriksdotter et al., 2018). To further investigate factors that could affect NGF-release or cell survival, exposure of NGF-producing cells to CSF from AD patients had a significantly reduced effect on NGF-release as compared to CSF from non-demented patients with subjective cognitive complaints (Eriksdotter et al., 2018).

Hence, the functionality of the ECB-NGF can be vulnerable to released mediators from activated glial cells, modulating the efficiency of the encapsulated NGF-releasing cells during longer duration of having devices in the brain. The variation of the patient’s clinical data, treatments and history of the diseases, could provide a milieu where the encapsulated cells might get affected. Therefore, disease conditions of the human brain play an important role in the function of encapsulated cells.

## Future of ECB-Neurotrophic Factor Therapy With NGF and BDNF

The establishment of ECB-NGF delivery provides a good model for having another cell-mediated therapy of BDNF for AD and other neurodegenerative disorders. Neurotrophins, both NGF and BDNF, improve cognition and hippocampal neuron viability and are reported to be reduced in AD brain.

The hippocampus represents a region, which remains neurogenic in the adult and recent data suggest that as many as 1,500 neurons are born in the hippocampus every day (Spalding et al., 2013). Neurotrophic signaling, and in particular BDNF plays a pivotal role in hippocampal neurogenesis, synaptic plasticity, and long-term potentiation, a proposed mechanism underlying memory formation at the level of the synapse (Acheson et al., 1995; Huang and Reichardt, 2001) In addition, BDNF show important modulatory effects at the synaptic level (Simonato et al., 2006). A study by Bolton et al. (2000) demonstrated selective different regulation mechanisms by BDNF at excitatory and inhibitory synapses. Moreover, various function of BDNF, neuronal and glial development and control of short- and long-lasting synaptic interactions that influence memory and cognition are included (Sasi et al., 2017; Kowianski et al., 2018).

Several studies have shown a decrease in BDNF in the hippocampus and in CSF during AD pathogenesis, suggesting that decreased BDNF signaling may contribute to the progression of hippocampal dysfunction in AD (Allen et al., 2013). Supporting this hypothesis, direct administration of BDNF, viral overexpression of BDNF or BDNF secreted from grafted stem cells improve age related cognitive decline and also rescue cognitive decline in a preclinical mouse models developing Aβ and tau pathologies (Nagahara et al., 2009b). The latter findings were observed in absence of any changes in Aβ or tau pathology in the brain. We have recently shown an increase in CSF BDNF in mild cognitive impairment patients as compared to AD patients suggestive of a complex relationship involving preclinical and clinical stages of the disease (Hjorth et al., 2013). Collectively, these data suggest that novel therapeutic strategies aimed at stimulating key neurotrophic signaling pathways or simultaneously ameliorating the toxic insult and stimulating neurodegeneration have the potential to deliver an efficient therapy to the patient. A possibility to explore feasibility of direct CNS delivery of the BDNF (in hippocampus) is the ECB technique. However, the question remains if there is a possibility to administer not only NGF but also BDNF using encapsulated technique for AD patients. First, experiments in animal models are needed to test this hypothesis.

In the ECB-NGF clinical trials, all AD patients were also on concomitant stable long-term ChEI-treatment. Hence, combinatorial treatment with neurotrophins delivery such as NGF and BDNF along with ChEIs in AD patients would be an interesting aspect to be looked upon in future (Mehta et al., 2012; Hampel et al., 2019).

The ECB-technique may be of interest for other brain diseases besides neurodegenerative disorders. In a very recent study, BDNF has been suggested as a therapeutic for chronic epilepsy (Iughetti et al., 2018). However, the delivery of BDNF for chronic epilepsy has met comparable difficulties since like NGF; BDNF does not cross the BBB. Therefore, similar to the ECB-NGF devices used in AD clinical trials, this approach was utilized to develop devices containing genetically engineered human cells lines to release BDNF. The purpose was to gain beneficial and efficiency effects of encapsulated ECB-BDNF in chronic epilepsy treatment (Falcicchia et al., 2018). The first ECB-BDNF delivery has recently been tested in a rat model, where after insertion of the ECB-BDNF device into the hippocampus of pilocarpine-treated rats, the frequency of spontaneous seizures was decreased. Additionally, an association with improved cognitive performance was shown. Importantly, behavioral evaluation of treated rats after long-term of BDNF delivery demonstrated normal behaviors such as general activity or sleep/wake patterns. Further, quantification and evaluation of histological analyses revealed neurological improvements related to BDNF-treatment in rats together with several anatomical changes, neuronal survival, and normalization of hippocampal volume (Falcicchia et al., 2018).

## ECB of Other Factors in Neurodegenerative Disorders

Besides BDNF and NGF, there are other neurotrophic factors, which can nourish, support and promote the survival and regeneration of the neurons. Among them CNTF, GDNF and neurturin have been extensively studied.

Parkinson’s disease (PD) is a neurodegenerative disorder that affects predominantly dopamine-producing (“dopaminergic”) neurons in substantia nigra. GDNF is the most efficient protective factor that promotes the survival and differentiation of dopaminergic neurons in experimental models of PD (Lin et al., 1993). Based on animal experiments and human studies, there is a need for safe, sustained and localized long-term delivery of GDNF into the basal ganglia of patients with PD (Emerich et al., 2014). Early rodent studies using encapsulated fibroblast cells and GDNF-secreting cells derived from retinal pigment epithelial cells in 6 months in rodents showed an enhanced dopaminergic function in animal models of PD (Lindner and Emerich, 1998). Compared to the practical, societal and ethical issues that have been associated with other transplantation approaches this method appeared to be promising (Sajadi et al., 2006; Huang et al., 2014; Garbayo et al., 2016). Even now, after two decades with collecting detailed knowledge of the approach of using GDNF as a therapeutic agent for PD, the main factors determining GDNF production remains obscure and the question whether GDNF can effectively be used as a therapeutic agent for PD remains unclear (d’Anglemont de Tassigny et al., 2015).

Huntington’s disease (HD) is an inherited disease that causes a progressive degeneration of nerve cells in the basal ganglia. One of the most striking physiological characteristics of HD is the loss of neurons in striatum, a component of the basal ganglia system that organizes motor movement and cognition. In recent years, there has been considerable research on neurotrophic factors and their effects on HD. Among the neurotrophic factors, CNTF has been studied in different models of HD as well as in non-human primates (Emerich et al., 1997a,b; Mittoux et al., 2000). ECB-CNTF has also been tested in one clinical trial (ClinicalTrials.gov Identifier: NCT01949324). However, in contrast to partly positive results from other neurodegenerative disorders that used ECB implants as a therapeutic approach, the clinical trial with CNTF-secreting encapsulated cells did not improve clinical symptoms. Investigation of the capsules and histopathological studies revealed that the treatment was not effective, suggesting poor distribution of CNTF from the ventricles and degeneration of the encapsulated cells (Bachoud-Levi et al., 2000; Bloch et al., 2004).

The encapsulated biodelivery technique has also been used to harbor cells that produce meteorin in animal studies, where the ECB was implanted into the rodent striatum using an animal model for HD (Tornoe et al., 2012). Meteorin is widely expressed in CNS. It is involved in glial cell differentiation and promotes axonal extension in small and intermediate neurons of basal ganglia nuclei whose degeneration is a hallmark of HD (Nishino et al., 2004). After treatment with ECB-meteorin for 4 weeks, the animals showed almost normal neurological performance and neuronal death in striatum was reduced. These results demonstrate new possibilities to further explore in neurological diseases with an excitotoxic component, such as HD (Tornoe et al., 2012). Another avenue to further investigate is the use of small molecules that can activate TrkA or p75-receptors to inhibit degeneration of neurons affected in HD (Simmons et al., 2017), as previously reported in AD (Simmons et al., 2014).

Since tau pathology correlates well with disease severity in AD and develops within the brain in anatomical connected regions, development of similar strategies using ECB is of interest. The spread of tau pathology has been suggested to occur in a prion-like manner from region to region (Jucker and Walker, 2013) suggesting that the extracellular pool of tau plays an important role in the progression of tauopathy in the brain. Indeed, high levels of anti-tau antibodies, administered intra-ventricularly via minipumps, have shown a pronounced effect on tau pathology development in an AD-like animal model, suggesting that selective targeting of extracellular tau in the brain has an impact on tau pathology development (Yanamandra et al., 2017). Proteases such as high temperature requirement A (HtrA) are involved in the protein quality control and known to degrade protein aggregations. In a recent report, human HTRA1 was shown to degrade aggregated and fibrillar tau, another pathological hallmark in AD (Poepsel et al., 2015). Interestingly, cell culture and biochemical experiments have revealed that HTRA1 can cleave both monomeric and aggregated tau. Most importantly, HTRA1 is secreted and active in the extracellular space, making it an example of a suitable candidate as an extracellular tau degrading protease. This feature of HTRA1 can be exploited, expressed and secreted using an EC biodelivery implant, to target extracellular tau.

## Conclusion

Alzheimer’s disease is a devastating neurodegenerative disease, and there is currently no effective therapy available. Thus, several proposals to explore radically new concepts for a future therapeutic strategy against AD are needed. The strong supportive preclinical data on both recent clinical data with neurotrophic factor cell therapy in AD patients and in the literature indicate that stimulation of neurotrophic signaling has a therapeutic potential for AD.

With regard to the difficulties of growth factors such as NGF or BDNF that do not significantly penetrate the BBB, the clinical utility requires invasive neurosurgical procedures. Searching literature and clinical trials using neurotrophins suggest multifunctional approaches to achieve optimal efficacy of the NGF/BDNF producing cells targeted to their optimal biologically relevant brain region. However, further development of therapeutic platforms such as the ECB and its efficacy in terms of cognitive outcomes is needed in AD and other neurodegenerative diseases. As an additional option or in combination with the ECB technique, synthetic modulators of NGF or BDNF signaling can be used to stimulate neurotrophic signaling in AD. The advancement of AD pathology includes multiple and concomitant processes. These processes can be partially normalized by the administration of NGF. Therefore, a combined treatment with these neurotrophins would be an attractive therapeutic approach for AD neurodegeneration.

## Author Contributions

ME initiated the manuscript and the idea, and finalized the manuscript. All authors contributed with design of manuscript and discussed content. SM and HB drafted the manuscript. All authors evaluated and reviewed the manuscript.

## Conflict of Interest Statement

The authors declare that the research was conducted in the absence of any commercial or financial relationships that could be construed as a potential conflict of interest.
